# Genome-wide association study of powdery mildew resistance in cultivated soybean from Northeast China

**DOI:** 10.3389/fpls.2023.1268706

**Published:** 2023-11-02

**Authors:** Yongsheng Sang, Hongkun Zhao, Xiaodong Liu, Cuiping Yuan, Guangxun Qi, Yuqiu Li, Lingchao Dong, Yingnan Wang, Dechun Wang, Yumin Wang, Yingshan Dong

**Affiliations:** ^1^ College of Agronomy, Jilin Agricultural University, Changchun, Jilin, China; ^2^ Soybean Institute, Jilin Academy of Agricultural Sciences, Changchun, Jilin, China; ^3^ Crop Germplasm Institute, Jilin Academy of Agricultural Sciences, Changchun, Jilin, China; ^4^ Department of Plant, Soil and Microbial Sciences, Michigan State University, East Lansing, MI, United States

**Keywords:** soybean, powdery mildew, single nucleotide polymorphism, GWAS, candidate genes

## Abstract

Powdery mildew (PMD), caused by the pathogen *Microsphaera diffusa*, leads to substantial yield decreases in susceptible soybean under favorable environmental conditions. Effective prevention of soybean PMD damage can be achieved by identifying resistance genes and developing resistant cultivars. In this study, we genotyped 331 soybean germplasm accessions, primarily from Northeast China, using the SoySNP50K BeadChip, and evaluated their resistance to PMD in a greenhouse setting. To identify marker-trait associations while effectively controlling for population structure, we conducted genome-wide association studies utilizing factored spectrally transformed linear mixed models, mixed linear models, efficient mixed-model association eXpedited, and compressed mixed linear models. The results revealed seven single nucleotide polymorphism (SNP) loci strongly associated with PMD resistance in soybean. Among these, one SNP was localized on chromosome (Chr) 14, and six SNPs with low linkage disequilibrium were localized near or in the region of previously mapped genes on Chr 16. In the reference genome of Williams82, we discovered 96 genes within the candidate region, including 17 resistance (R)-like genes, which were identified as potential candidate genes for PMD resistance. In addition, we performed quantitative real-time reverse transcription polymerase chain reaction analysis to evaluate the gene expression levels in highly resistant and susceptible genotypes, focusing on leaf tissues collected at different times after *M. diffusa* inoculation. Among the examined genes, three R-like genes, including *Glyma.16G210800*, *Glyma.16G212300*, and *Glyma.16G213900*, were identified as strong candidates associated with PMD resistance. This discovery can significantly enhance our understanding of soybean resistance to PMD. Furthermore, the significant SNPs strongly associated with resistance can serve as valuable markers for genetic improvement in breeding *M. diffusa*-resistant soybean cultivars.

## 1 Introduction

Cultivated soybean (*Glycine max L.*) is a significant oil crop worldwide, providing essential food, oil, and protein (Hartman et al., 2011). Powdery mildew (PMD), caused by the pathogen *Microsphaera diffusa* Cooke & Peck, is a widespread fungal disease inflicting substantial economic losses in soybean production regions, such as Brazil, Japan, northeast India, south China, Australia, and parts of the USA (Dunleavy, 1976; Leath and Carroll, 1982; Gonçalves et al., 2002; Takamatsu et al., 2002; Mctaggart et al., 2012; Baiswar et al., 2016; Li et al., 2016). PMD development is favored by moderate rainfall, high humidity, and low temperatures (Phillips, 1984). Mignucci and Boyer (1979) discovered 18°C to be favorable for PMD development on susceptible cultivars, while Alves et al. (2009) reported that temperatures around 23°C and 24°C favored PMD intensity progress in Conquista and Suprema cultivars, respectively. Hence, the optimal temperature range for PMD development is 18°C to 24°C. Below 15°C and above 30°C, the infection severity may decrease (Mignucci and Boyer, 1979; Phillips, 1984; Alves et al., 2009). PMD symptoms on susceptible plants include white powder patches on leaf surfaces, chlorosis, yellow islands, rusty stains, defoliation, and a combination of these (Grau, 2006; Mctaggart et al., 2012). PMD often reduces soybean yield by 30–40% and can even result in total loss in susceptible cultivars during epidemic years (Gonçalves et al., 2002; Jun et al., 2012).

Host plant resistance is the most effective measure to reduce PMD damage (Ramalingam et al., 2020). Three alleles at the *Rmd* locus—*Rmd*, *Rmd-c*, and *rmd*—determine soybean’s response to PMD (Lohnes and Bernard, 1992). Although the *Rmd-c* allele provides resistance to PMD throughout the soybeans’ entire growth cycle, the *Rmd* gene governs adult plant resistance to PMD (Mignucci and Lim, 1980). In contrast, soybean plants carrying the homozygous recessive allele *rmd* are susceptible throughout their life cycle (Lohnes and Bernard, 1992). The *Rmd-c* gene of Williams isoline L76-1988 is located on soybean classical LG19 equivalent to chromosome (Chr) 16 between Rps2 and Rj2, with genetic distances of 2.3 cM and 1.9 cM, respectively (Lohnes et al., 1993; Polzin et al., 1994). *Rmd_PI243540* from cultivated soybean PI 243540 is situated within a 10.9 cM region flanked by the single-nucleotide polymorphisms (SNPs) marker BARC-021875-04228 and the simple sequence repeat (SSR) marker Sat_224 (Kang and Mian, 2010). *Rmd_PI567301B* in cultivar PI567301B is located within a 1.4 cM region flanked by the SSR markers BARCSOYSSR_16_1298 and BARCSOYSSR_16_1272 (Jun et al., 2012). *Rmd_V97-3000* in cultivar V97-3000 is located between two SSR markers Satt547 and Sat_396, at distances of 3.8 cM and 3.9 cM, respectively (Wang et al., 2013). Similarly, *Rmd_B3* in cultivar B3 is located between SSR markers GMES6959 and Satt_393, with distances of 7.1 cM and 4.6 cM, respectively. Furthermore, *Rmd_B13* in cultivar B13 is delimited to a 188.06 kb region harboring 28 genes (Jiang et al., 2019). Recently, the PMD adult plant resistance gene *Rmd_ZH24* from cultivar ZH24 was precisely located within a 32.8-kb genomic interval region delimited by the markers Gm16_428 and InDel14 on Chr16. To date, PMD resistance (R) genes from seven donor soybean cultivars or lines have all been mapped to the end of Chr16 (Zhou et al., 2022).

Genome-wide association studies (GWAS) have become a powerful alternative to linkage mapping for analyzing complex trait variations at the genomic level, utilizing ancient recombination events at the population level (Zhu et al., 2008). GWAS significantly improves the precision and accuracy of marker-phenotype associations compared to linkage analysis with biparental mapping populations. In soybean, GWAS has been utilized to identify markers linked to various disease resistance traits, including soybean cyst nematode resistance (Chang et al., 2016; Zhang et al., 2016; Tran et al., 2019; Shi et al., 2021), sudden death syndrome (Zhang et al., 2015; Chang et al., 2016), Sclerotinia stem rot (Wei et al., 2015; Wei et al., 2017; Jing et al., 2021), Soybean mosaic virus (Chang et al., 2016; Che et al., 2017), white mold (Bastien et al., 2014; Wen et al., 2018), root knot nematode (Alekcevetch et al., 2021), Phytophthora root rot (Chang et al., 2016; Li et al., 2022), southern root knot nematode (Passianotto et al., 2017), and brown stem rot (Chang et al., 2016; Rincker et al., 2016), as well as resistance to bacterial pustule, Diaporthe stem canker, soybean rust, reniform nematode, Bean pod mottle virus, and Peanut mottle virus (Chang et al., 2016). Despite identifying PMD R-genes through bi-parental crosses (Kang and Mian, 2010; Jun et al., 2012; Wang et al., 2013; Jiang et al., 2019; Zhou et al., 2022), GWAS is rarely used to investigate traits associated with soybean PMD resistance. Therefore, this study aimed to (i) identify genes linked to PMD resistance using GWAS and (ii) explore potential genes at GWAS-identified loci through differential gene expression analysis. The results can enhance our understanding of the genetic control of PMD resistance and provide potential molecular markers for breeding PMD-resistant soybean cultivars against *M. diffusa*.

## 2 Results

### 2.1 Phenotypic analysis of PMD resistance

**Figure 1:**
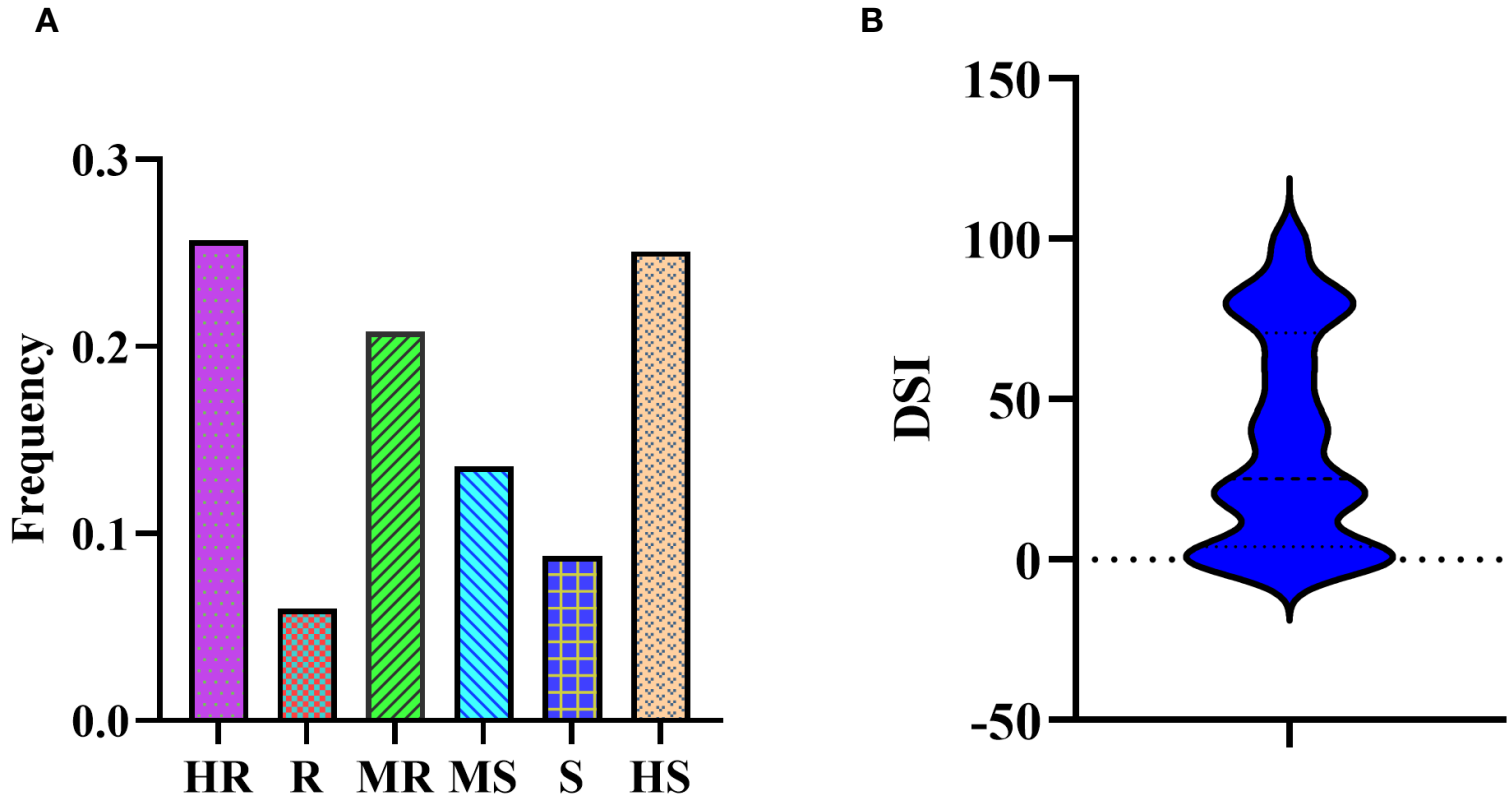
Disease severity of 331 SGAs to *M.diffusa*. **(A)** Bio-assay phenotyping results of 331 SGAs for PMD resistance. **(B)** Bio-assay phenotypic distribution results of 331 SGAs for PMD resistance.

In the association mapping population, we observed a substantial variance in PMD resistance based on the disease severity index (DSI) (**Supplementary Table S1**). The DSI values for the 331 soybean germplasm accessions (SGAs) ranged from 0 to 100, with a mean of 36.92, and followed a reverse normal distribution. Among the 331 SGAs evaluated, 85 accessions were highly resistant (HR), 20 were resistant (R), 69 were moderately resistant (MR), 45 were moderately susceptible (MS), 39 were susceptible (S), and 83 were highly susceptible (HS) (**Figures 1A, B**).

### 2.2 Quality control and linkage disequilibrium decay

**Figure 2:**
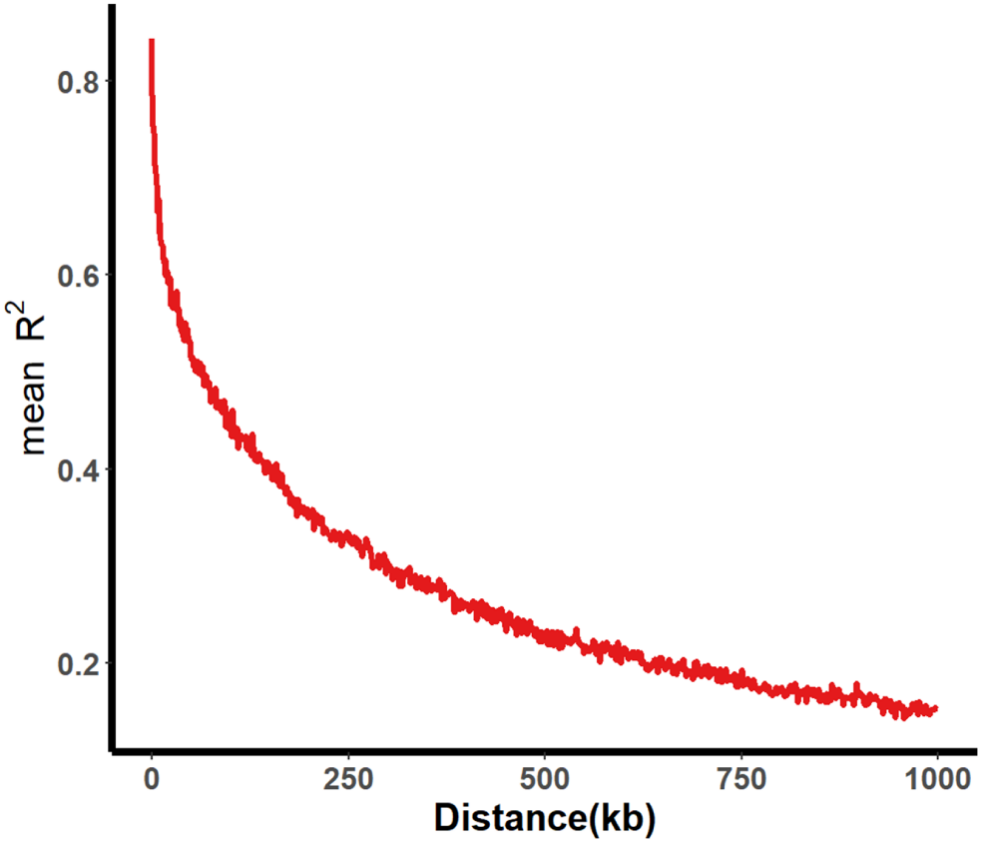
Average LD decay of 331 SGA associated populations. The average LD decay rate was estimated as r^2^ employing all pairs of SNP markers located within a 1000 kb physical distance in euchromatic and heterochromatic regions in 331 SGAs.

A total of 331 SGAs were genotyped using the SoySNP50K BeadChip, resulting in the characterization of profiles for 52,041 single nucleotide polymorphisms (SNPs). After filtering SNPs with a minor allele frequency of less than 5% in at least 80% of genotypes, 30,602 high-quality SNPs were obtained, providing widespread coverage across the entire soybean genome. Population structure analysis and association mapping were performed using these high-quality SNPs. Within a 1000 kb window, pairwise linkage disequilibrium (LD) was estimated, and the LD decay rate, measured by the point at which the correlation coefficient (r^2^) dropped to half of its maximum value, was determined to be 109 kb at r^2^ = 0.422 (**Figure 2**). The LD decay observed was lower than previously reported values for improved lines (233 kb) and landraces (187 kb) (Wen et al., 2015). This difference may be attributed to the involvement of fewer genotypes in the two panels, as the same BeadChip was used for genotyping.

### 2.3 Analysis of population structure of 331 SGAs

**Figure 3:**
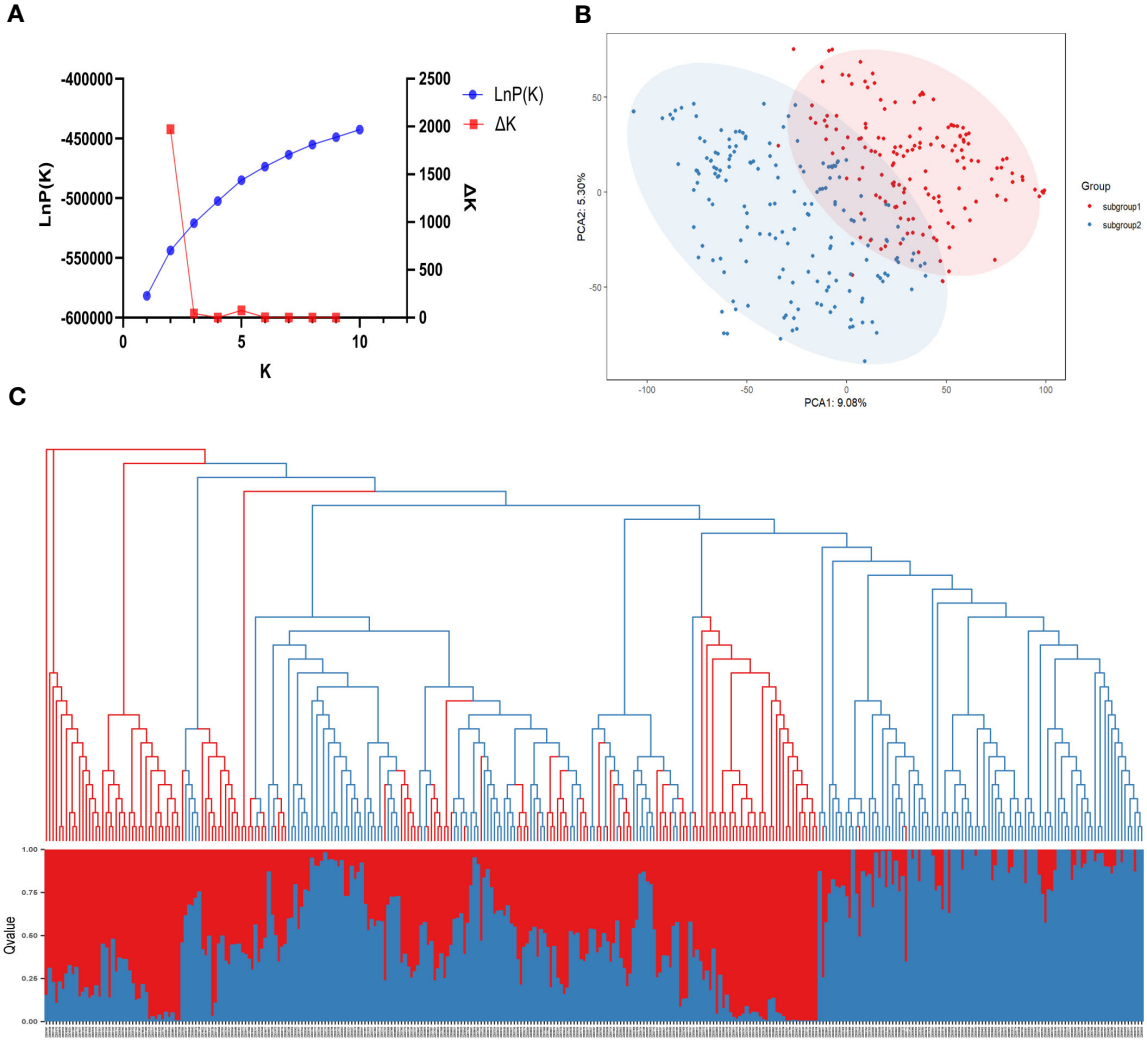
Population structure analysis of 331 SGAs. **(A)** The average of LnP(k) and Delta k values when k varies from 1 to 10. **(B)** In a two-dimensional scatter plot of PCA, subgroups 1 and 2 are represented by the red and blue dots, respectively. **(C)** Combination map of population structure and neighbor-joining tree for 331 SGAs, with each segment representing the percentage of individuals in the population. Grouped by population structure.

The population structure of the 331 SGAs was analyzed using STRUCTURE 2.3.4 software (Pritchard et al., 2000) based on 1643 unlinked SNPs. A sharp peak of Delta K at K=2 (**Figure 3A**) indicated the presence of two sub-populations, designated as clusters Q1 and Q2. Among the 331 SGAs, 155 were assigned to the Q1 sub-population, comprising 56 from Hei Long Jiang Province (HLJ), 60 from Ji Lin Province (JL), 31 from Liao Ning Province (LN), 7 from Inner Mongolia (IM), and 1 from Bei Jing (BJ). The Q2 sub-population consisted of 176 SGAs, including 63 from HLJ, 87 from JL, and 26 from LN (**Figure 3C**; **Supplementary Table S2**). A Q-matrix was obtained and utilized for association mapping after determining the optimal K value. Principal component analysis (PCA) and phylogenetic tree analysis of the 331 SGAs confirmed the clustering patterns predicted by the STRUCTURE analysis (**Figures 3B, C**). These results indicated a subpopulation structure among the 331 SGAs, and the Q matrix could be incorporated as a covariate to reduce the false positive rate in the GWAS model.

### 2.4 GWAS for PMD resistance

**Figure 4:**
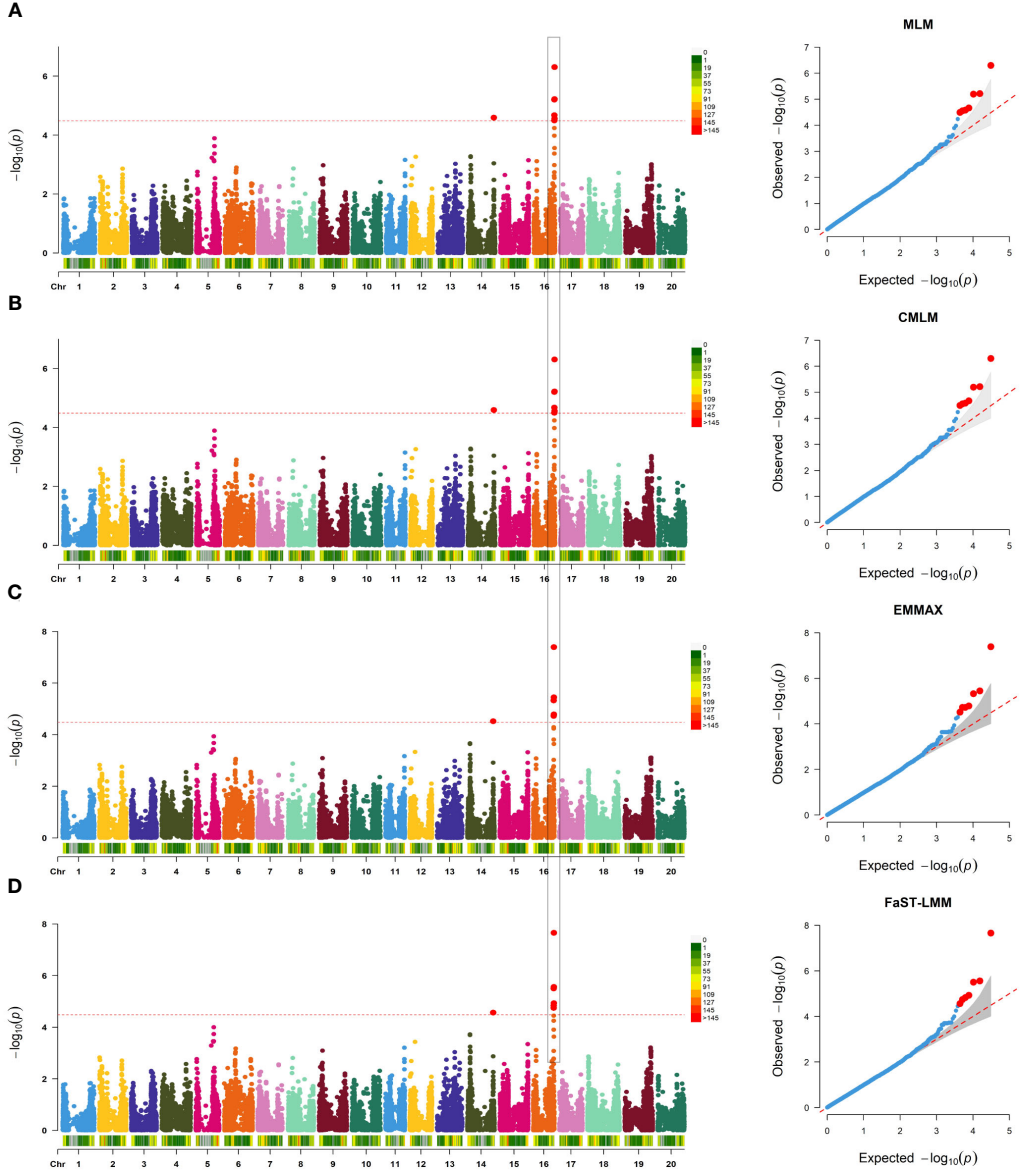
GWAS of PMD in 331 SGAs. **(A)** Manhattan plot and Q-Q plot generated from genome-wide analysis of PMD resistance in 331 SGAs using MLM(Q+K). Negative log10 P-values from a genome-wide scan are displayed against the position on each soybean chromosome. The horizontal red dotted line indicates the Bonferroni test threshold as 1/total (-log10P=4.49). **(B)** Manhattan plot and Q-Q plot generated from genome-wide analysis of PMD resistance in 331 SGAs using CMLM (Q+K), as in **(A)**. **(C)** Manhattan plot and Q-Q plot generated from genome-wide analysis of PMD resistance in 331 SGAs using EMMAX (Q+K), as in **(A)**. **(D)** Manhattan plot and Q-Q plot generated from genome-wide analysis of PMD resistance in 331 SGAs using FaST-LMM (K), as in A.

**Figure 5:**
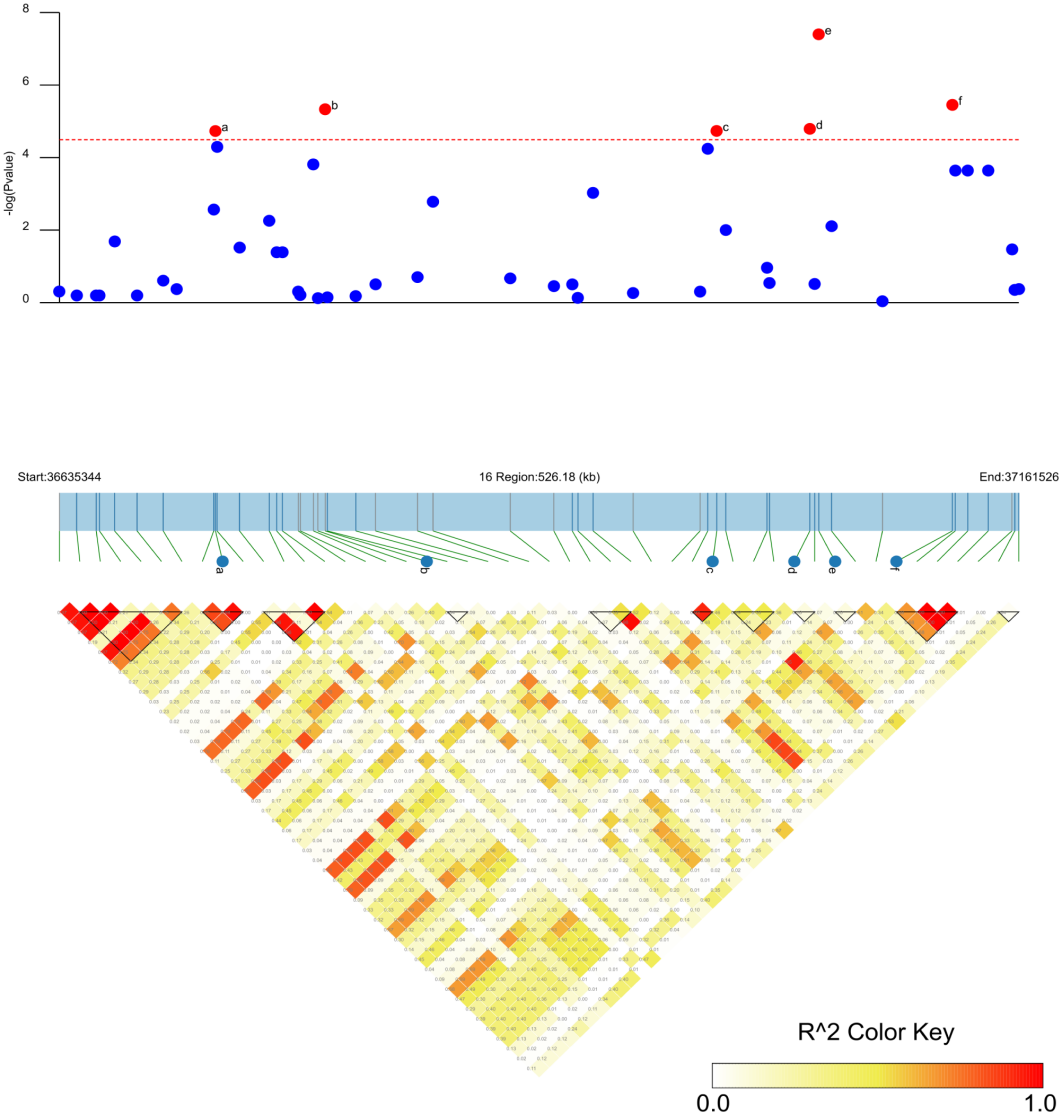
Candidate regions of the six significant SNP loci associated with PMD in soybean. The -log10 P-values of the SNPs from the PMD GWAS are displayed in the top panel for the physical location of a given chromosome region. The bottom panel represents the horizontal range of LD in the area calculated using R^2^, and the color key shows R^2^ values. The horizontal red dotted line represents a significant threshold for GWAS (-log10(p) >4.49). a: significant SNP ss715624888; b: significant SNP ss715624901. c: significant SNP ss715624926; d: significant SNP ss715624931. e: significant SNP ss715624933; f: significant SNP ss715624939.

**Figure 6:**
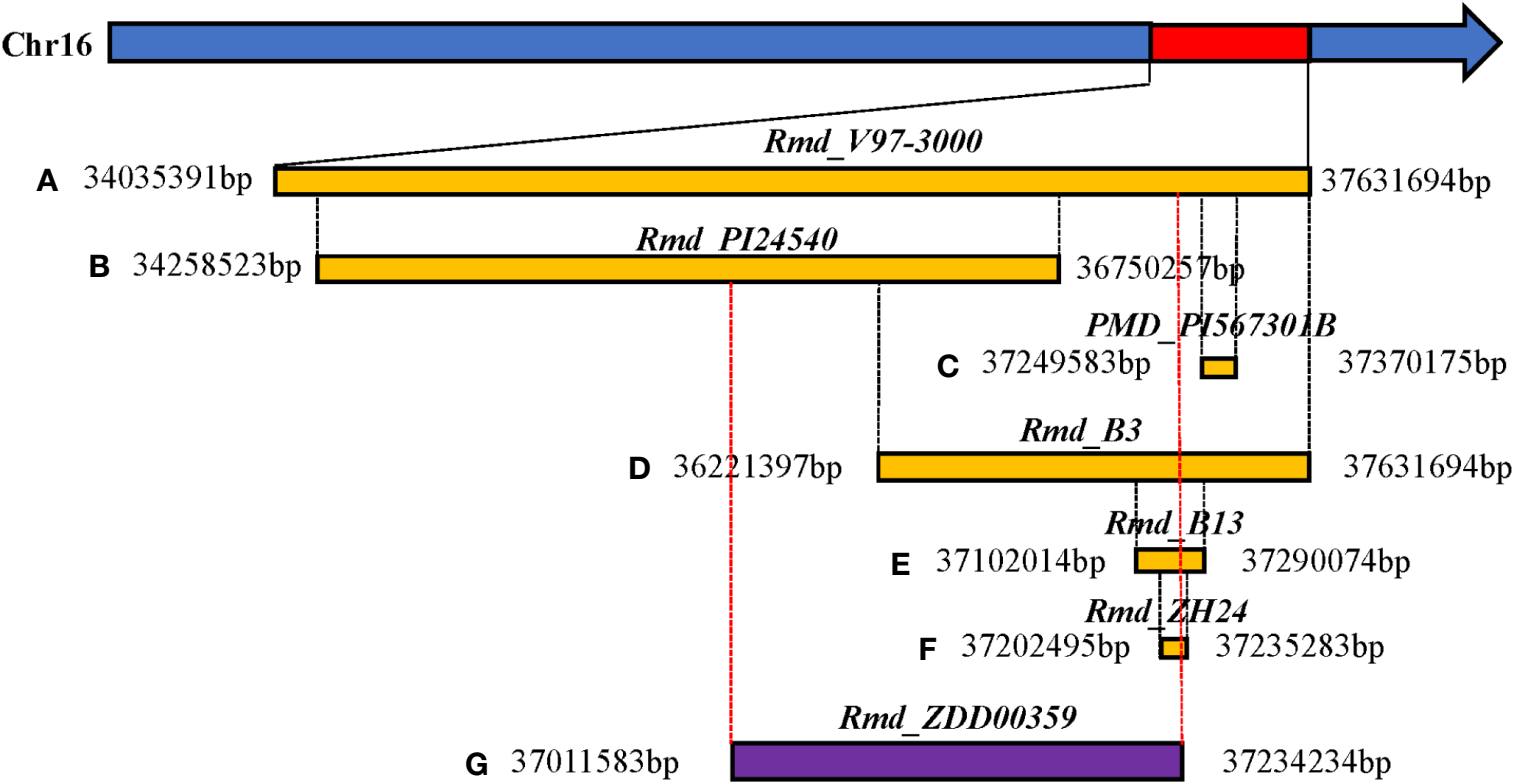
Comparison of physical location between candidate regions of the large-effect marker (ss715624933) and reported resistance genes to PMD in soybean. **(A)** Relative physical location of the PMD resistance gene *Rmd_V97-3000*. **(B)** Relative physical location of the PMD resistance gene *Rmd_PI24540*. **(C)** Relative physical location of the PMD resistance gene *PMD_PI567301B*. **(D)** Relative physical location of the PMD resistance gene *Rmd_B3*. **(E)** Relative physical location of the PMD resistance gene *Rmd_B13*. **(F)** Relative physical location of the PMD resistance gene *Rmd_ZH24*. **(G)** Relative physical location of the PMD resistance gene *Rmd_ZDD00359*. *Rmd_ZDD00359*: The linkage region of the six significant SNP loci on Chr16 (ss715624888-60.2kb-ss715624901-214.6kb-ss715624926-51.1kb-ss715624931-4.8kb-ss715624933-73.3kb-ss715624939).

SNP-trait associations for DSI were investigated using four models: factored spectrally transformed linear mixed models (FaST-LMM), compressed mixed linear model (CMLM), mixed-model association eXpedited (EMMAX), and efficient mixed linear model (MLM). Quantile-quantile plots (Q-Q plots) of the four models showed initial consistency between observed and expected P values, with significant deviation beginning from the expected P value as the -log10P value increased to approximately 3.5 (**Figure 4**). The four models demonstrated very effective control of error associations. The significance threshold with Bonferroni correction was set at -log10(1/30,602) = 4.49. From the greenhouse evaluations, we identified seven strongly associated SNPs with DSI (**Table 1**, **Figure 4**): one SNP on Chr14 (ss715619284) and six on Chr16 (ss715624888-60.2kb-ss715624901-214.6kb-ss715624926-51.1kb-ss715624931-4.8kb-ss715624933-73.3kb-ss715624939). Among these, ss715624933 exhibited the highest significance across all four GWAS models, followed by ss715624939 and ss715624901. The pairwise LD of the six significant SNPs on Chr16 was relatively low, indicating no tight linkage between them (**Figure 5**). Comparing the locations of the significant SNPs identified in this study with published R-genes from previous bi-parental mapping results, ss715624888 and ss715624901 were found in the overlapped region of *Rmd_B3* and *Rmd_V97-3000*, while the other four SNPs were located in front of *Rmd_B3*, but not within the genomic regions of *Rmd_B13*, *Rmd_PI567301B*, and *Rmd_ZH24* (**Figure 6**).

**Table 1:** List of significant SNPs detected by different statistic models.

Year	Method	SNP	Physical position		Significant region		-log10(P)
			Chr.	Position	Start	End	
2019	MLM	ss715624933	16	37051712	36942712	37160712	6.3
		ss715624939	16	37125034	37016034	37234034	5.22
		ss715624901	16	36781107	36672107	36890107	5.2
		ss715624888	16	36720932	36611932	36829932	4.67
		ss715619284	14	46661760	46552760	46770760	4.59
		ss715624931	16	37046875	36937875	37155875	4.56
		ss715624926	16	36995747	36886747	37104747	4.5
	CMLM	ss715624933	16	37051712	36942712	37160712	6.3
		ss715624939	16	37125034	37016034	37234034	5.22
		ss715624901	16	36781107	36672107	36890107	5.2
		ss715624888	16	36720932	36611932	36829932	4.67
		ss715619284	14	46661760	46552760	46770760	4.59
		ss715624931	16	37046875	36937875	37155875	4.56
		ss715624926	16	36995747	36886747	37104747	4.5
	EMMAX	ss715624933	16	37051712	36942712	37160712	7.39
		ss715624939	16	37125034	37016034	37234034	5.45
		ss715624901	16	36781107	36672107	36890107	5.33
		ss715624931	16	37046875	36937875	37155875	4.79
		ss715624926	16	36995747	36886747	37104747	4.73
		ss715624888	16	36720932	36611932	36829932	4.73
		ss715619284	14	46661760	46552760	46770760	4.52
	FastLMM	ss715624933	16	37051712	36942712	37160712	7.66
		ss715624939	16	37125034	37016034	37234034	5.56
		ss715624901	16	36781107	36672107	36890107	5.5
		ss715624931	16	37046875	36937875	37155875	4.93
		ss715624888	16	36720932	36611932	36829932	4.83
		ss715624926	16	36995747	36886747	37104747	4.75
		ss715619284	14	46661760	46552760	46770760	4.56

### 2.5 Prediction of candidate genes underlying PMD resistance

**Figure 7:**
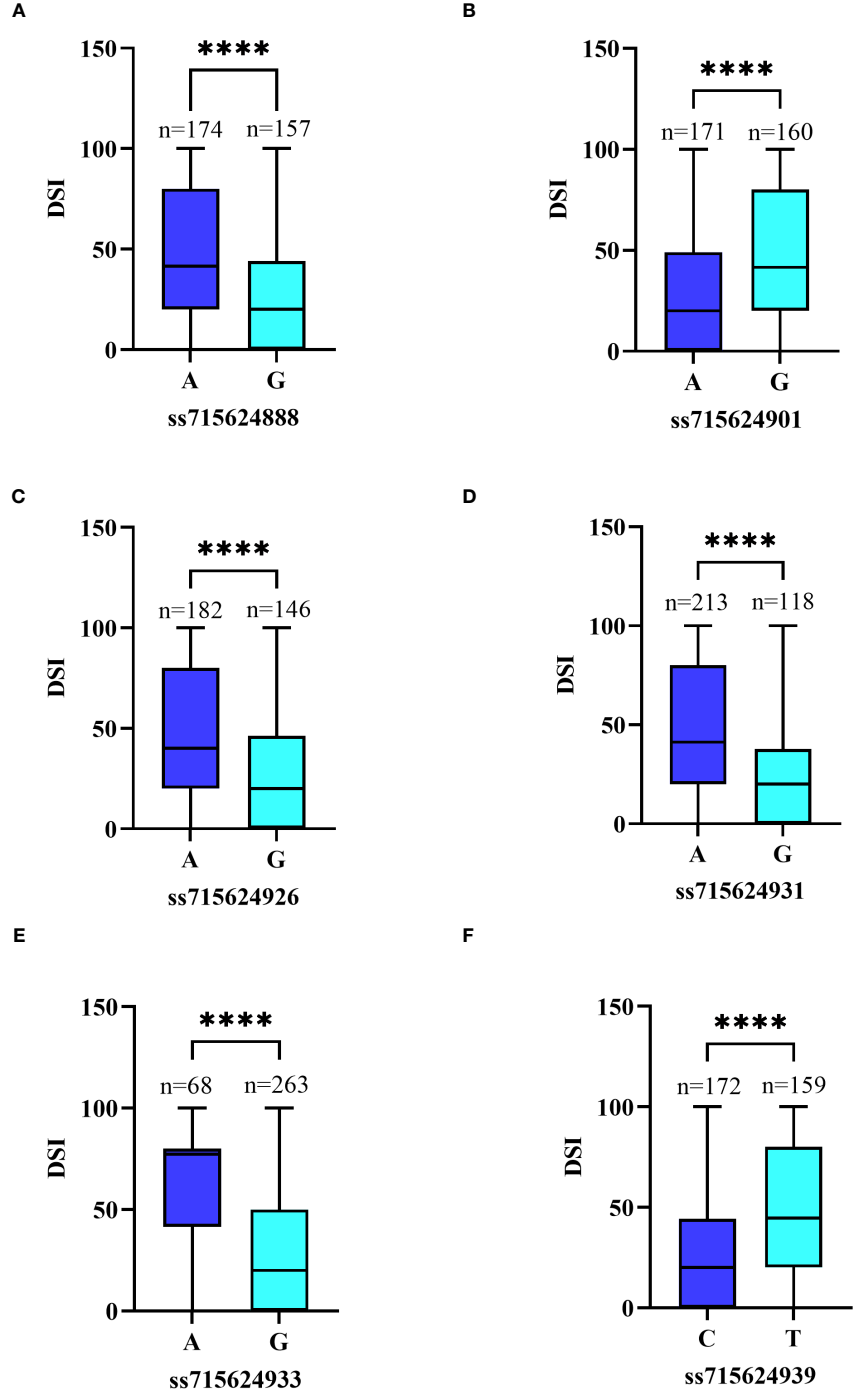
Differences in PMD resistance between accessions carrying different alleles. **(A)** Allele effect of PMD marker ss715624888 in 331 SGAs. **(B)** Allele effect of PMD marker ss715624901 in 331 SGAs. **(C)** Allele effect of PMD marker ss715624926 in 331 SGAs. **(D)** Allele effect of PMD marker ss715624931 in 331 SGAs. **(E)** Allele effect of PMD marker ss715624933 in 331 SGAs. **(F)** Allele effect of PMD marker ss715624939 in 331 SGAs. The effects of different alleles were statistically analyzed using an unpaired two-tailed Mann–Whitney’s t-test. **** denotes the statistical significance level at P < 0.0001.

We focused on six significant SNPs on Chr16: ss715624933 (Gm16_37,051,712, MAF=0.207), ss715624939 (Gm16_37,125,034, MAF=0.486), ss715624901 (Gm16_36,781,107, MAF=0.483), ss715624931 (Gm16_37,046,875, MAF=0.356), ss715624926 (Gm16_36,995,747, MAF=0.446), and ss715624888 (Gm16_36,720,932, MAF=0.474). Soybean plants carrying the favorable allele (GG) on ss715624933, ss715624888, ss715624926, and ss715624931 showed significantly higher PMD resistance (mean DSI=30.10, 27.39, 27.58, and 22.38, respectively) than those carrying the unfavorable allele (AA) (mean DSI=63.28, 45.61, 45.07, and 45.05, respectively) (**Figures 7A–D**). Similarly, soybeans carrying the favorable allele (AA) on ss715624901 exhibited significantly higher PMD resistance (mean DSI=28.19) than those carrying the adverse allele (GG) (Mean DSI=46.35) (**Figure 7E**). Soybeans carrying the favorable allele (CC) on ss715624939 also displayed significantly higher PMD resistance (mean DSI=28.04) than those carrying the alternative allele (AA) (mean DSI=46.53) (**Figure 7F**). Due to the low LD between the six significant SNP loci, we focused on a 622.1kb region (from 109kb before ss715624888 to 109kb after ss715624939) and performed candidate gene prediction based on gene models of the cultivated soybean genome assembly version Glyma.Wm82.a2.v1. Within this region, we identified a total of 96 putative causal genes, of which 17 genes possessed the Toll-interleukin receptor (TIR)-nucleotide binding site (NBS)-leucine-rich repeat (LRR) domain, known for its significance in soybean disease resistance (**Supplementary Table S3**). The 17 R-like genes were annotated using the Gene Ontology (GO, https://www.ebi.ac.uk/QuickGO/) and the eukaryotic orthologous groups (KOG, https://www.ncbi.nlm.nih.gov/Structure/cdd/cddsrv.cgi?uid=KOG1493) databases. Most of these genes displayed similar functional descriptions, including protein kinase activity, innate immune response, ADP binding, apoptosis, protein phosphorylation, protein binding, nucleic acid binding, signal transduction, and other biological and metabolic processes (**Supplementary Table S4**). Additionally, they participated in plant cell signal transduction mechanisms by producing leucine-rich repeat proteins, serine or threonine protein kinases, and some proteins containing F-box and apoptotic ATPase (**Supplementary Table S5**). Moreover, *Glyma.16g208100*, *Glyma.16g208200*, and *Glyma.16g208300* were identified to play roles in plant defense mechanisms by producing arylacetamide deacetylase (**Supplementary Table S5**). These three genes shared similar functional descriptions, including pollen tube growth, hydrolase activity, carboxylic ester hydrolase activity, and other catabolic and metabolic processes (**Supplementary Table S4**). Based on these findings, we considered these 17 R-like genes and the trio of carboxyesterase 18 genes as potential candidate genes.

### 2.6 Expression profiling for candidate genes

**Figure 8:**
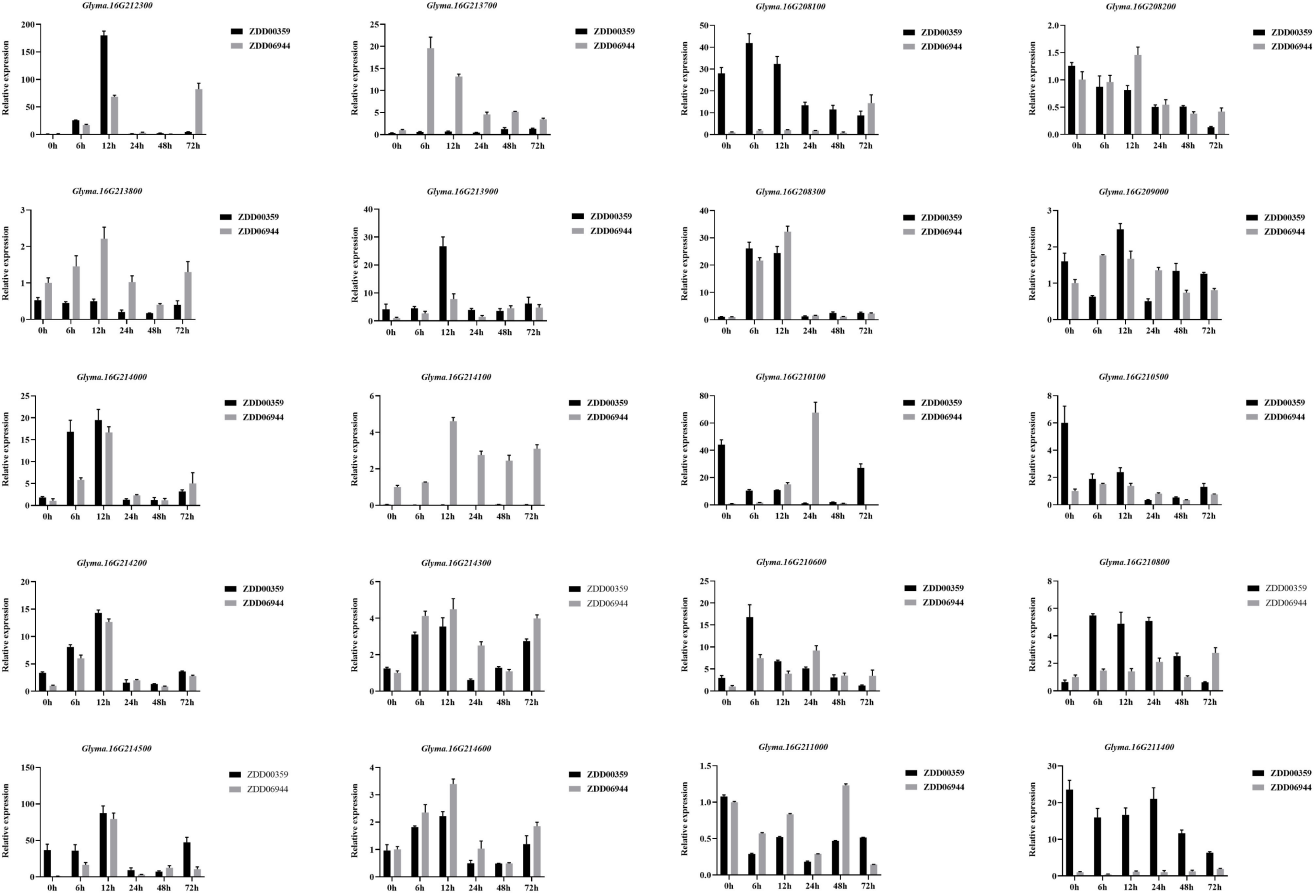
Relative expression levels of candidate genes of *Rmd_ZYDD00359* in ZYDD06944 (HS) and ZYDD00359 (HR). ZYDD06944 and ZYDD00359 seedlings were cultivated for 10 d, followed by spraying *M. diffusa* spore suspension (1×10^5^ cfu/ml) and examined at 0, 6, 12, 24, 48, and 72 h post-inoculation. Values are presented as means ± SEs (n ≥ 3).

We analyzed the expression patterns of 20 candidate genes in ZDD06944 (which carries unfavorable alleles at ss715624888, ss715624901, ss715624926, ss715624931, ss715624933, and ss715624939 loci and is considered susceptible, HS.) and ZDD00359 (which carries favorable alleles at ss715624888, ss715624901, ss715624931, ss715624933, and ss715624939 loci and is considered resistant, HR) using real-time reverse transcription polymerase chain reaction (qRT-PCR) analysis (**Figure 8**). Among the 20 genes, three genes (*Glyma.16G210800*, *Glyma.16G212300*, and *Glyma.16g213900*) displayed differential expression between ZDD06944 and ZDD00359, and were up-regulated in the highly resistant accession ZDD00359 after *M. difusa* treatment. In ZDD00359 (HR), the expression patterns of these three putative candidate genes significantly increased at 6 and 12 h after treatment, with peak expression observed at 12 h. *Glyma.16G212300* also showed increased expression at 6 h, reaching the maximum value (approximately 216.8-fold) at 12 h, and then rapidly decreased at 24, 48, and 72 h. A comparable expression pattern was observed for *Glyma.16g213900*, where the expression level peaked (approximately 6.5-fold) at 12 h after treatment, followed by a rapid decrease. In contrast, the expression level of *Glyma.16G210800* in ZDD00359 (HR) reached the maximum value (approximately 8.56-fold) at 6 h after treatment and started to decrease at 48 h. Based on these expression patterns, we concluded that these three R-like genes were induced by *M. diffusa* and may play a role in the soybean’s disease defense mechanism.

## 3 Discussion

In this study, we identified a total of seven SNPs significantly associated with PMD resistance, and the LD analysis revealed that they were not tightly linked. Previous studies consistently identified the end of Chr16 as the location of all PMD R-genes in different soybean varieties or lines (PI567301B, V97-3000, PI243540, B13, B3, and ZH14). These gene regions were determined through a blast search of flanking markers based on the cultivated soybean reference genome (Williams 82.a2.v1) (**Figure 6**). Specifically, *Rmd_V97-3000* was located within a region of approximately 3.6 Mb, covering all other mapped R-genes. The regions of *Rmd_PI24540* differed from those of *Rmd_B1*, *Rmd_ZH14*, and *PMD_PI567301B*. Additionally, the regions of *Rmd_B1* and *Rmd_ZH14* were distinct from that of *PMD_PI567301B*. *Rmd_B13*’s location partly overlapped with the region of *Rmd_PI567301B*, while it was different from that of *Rmd*_PI24540. Previous studies have suggested that soybean PMD resistance sources, such as PI567301B, PI243540, and the cultivated variety CNS, may carry different R-genes based on map positions and verification tests of three markers (Jun et al., 2012). Combining the comparative analysis of the mapping results of these R-genes with our GWAS findings, it could be possible that three or more loci were involved in controlling PMD resistance. However, confirmation through experimental support, such as allelism tests, was required. Furthermore, if the sources mentioned above carried distinct R-genes, a gene pyramid composed of different R-genes could potentially enhance resistance to PMD. Nevertheless, our study demonstrated that GWAS analysis was an excellent tool for identifying the gene(s) underlying soybean PMD resistance. Moreover, the SNPs strongly linked to PMD resistance identified in this study were valuable for molecular marker-assisted selection (MAS).

GWAS signals are often challenging to interpret biologically, as they frequently reside in gene deserts or regions with multiple plausible causative genes (Nica et al., 2010). Examining differential gene expression patterns has been proposed as a promising method to understand GWAS signals biologically better (Emilsson et al., 2008). In this study, we identified a total of 96 putative genes within a 622.1 kb region, including 17 genes with the TIR-NBS-LRR domain crucial for disease resistance in plants. Among these, *Glyma.16G210800*, *Glyma.16G212300*, and *Glyma.16g213900* exhibited differential expression between ZDD06944 and ZDD00359, with up-regulated expression in the highly resistant accession ZDD00359 after *M. diffusa* infection. Previous studies have suggested that genes showing distinct expression patterns among different accessions tend to be associated with susceptibility or resistance results, either directly or indirectly. Conversely, genes with different expression dynamics across time may represent the general plant reactions to pathogen infections without necessarily conferring increased resistance (Calla et al., 2009). Therefore, these genes are considered as strong candidate genes. Jiang et al. (2019) discovered that nine of the 17 R-like genes in *Rmd_B13* exhibited differential expressions in resistant and susceptible parents. As the 622.1 kb candidate region partially overlapped with *Rmd_B13*, the nine R-like genes within the overlapped region were assayed by qRT-PCR in this experiment. Among them, *Glyma.16g213900* consistently demonstrated altered expressions after *M. diffusa* treatment, suggesting its potential role in regulating soybean PMD defense. However, another gene, *Glyma.16G212300*, located near the peak SNP, showed significantly up-regulated expression in the highly resistant accession ZDD00359 and deserved special attention. Future studies will focus on verifying the functional effects of these R-like genes and elucidating the molecular mechanisms underlying soybean PMD resistance.

Currently, a substantial number of plant disease-resistant genes have been identified, with approximately 80% belonging to the NBS-LRR gene family, which includes a central NBS domain and C-terminal LRR based on whether the N-terminal is homologous to TIR (Meyers et al., 1999; Bai et al., 2002; Cannon et al., 2002). Kang et al. (2012) reported the presence of 175 disease-resistant quantitative trait loci (QTLs) and 319 hypothetical NBS-LRR genes in soybean. Among these, 40 genes encoded NBS-LRR proteins, and 19 disease-resistant QTLs were clustered on Chr16. It is believed that gene families providing disease resistance may have clustered together through duplication and divergence of common ancestors (Ribas et al., 2011). Notably, PMD resistance loci contain clustered R-like genes, and nearby regions have been discovered to harbor disease R-genes or QTLs for resistance to biotic stressors in the soybean genome, such as a cluster of resistance gene analogs (Graham et al., 2002). For plant breeders, clustering these R-genes poses challenges in pyramiding and introgressing various resistance alleles into a single breeding line, especially when recombination suppression is present (Verlaan et al., 2011). Previous studies have demonstrated that some R-gene clusters act as natural pyramids of resistance genes against different pathogens. For example, certain Mi-1 homologs in NIL-Ol-4 have been identified to confer resistance to aphids, nematodes, and PMD (Seifi et al., 2011). Therefore, the efforts to identify soybean sources with multiple resistances to different causal pathogens hold great promise for enhancing soybean breeding programs.

## 4 Conclusions

In this study, GWAS identified seven SNPs significantly associated with PMD resistance. Three genes (G*lyma.16G210800*, *Glyma.16G212300*, and *Glyma.16g213900*) presented differential expression between highly susceptible and highly resistant accessions after *M. diffusa* infection, suggesting their potential as candidate genes. These PMD resistance-associated SNPs could serve as valuable markers for MAS in soybean breeding.

## 5 Materials and methods

### 5.1 Plant materials and inoculation

All 331 SGAs used in this study were provided by the National Genebank of China (Beijing, China) and maintained by the Soybean Institute of Jilin Academy of Agricultural Sciences (**Supplementary Table S1**). The SGAs mainly originated from Northeast China, comprising 127 bred varieties (lines) and 204 landraces (**Supplementary Table S6**). The greenhouse experiment was conducted at the Jilin Academy of Agricultural Sciences (Gongzhuling, China) in September 2019. Each SGA, along with resistant and susceptible controls, was sown in a 2-gallon plastic pot with 10 seeds, and after emergence, the seedlings were thinned to 5 plants. The experiment followed a complete block randomized design with three replications. For infection, soybean leaves from HS plants were used to obtain *M. diffusa* spores. The spores were then cleaned from the leaves of susceptible plants and sprayed evenly on each plant’s leaves at the V1 stage using a 1×10^5^ cfu/ml spore suspension until the top leaves were completely wet (Jun et al., 2012). The inoculated soybeans were maintained in the greenhouse with a temperature range of about 18–25°C and a photoperiod of 8 h night and 16 h light. Daily plant management was performed, and water spray was done twice a day at 8:00 AM and 5:00 PM to maintain leaf wetness for one week.

### 5.2 Resistance evaluation in greenhouse

Four weeks after inoculation, the disease response of leaves to PMD was evaluated using a modified criterion from Li et al. (2016). Each plant in the 2-gallon plastic pot was individually assessed on a scale of 0 to 5, where 0 indicated no foliar symptoms, 1 indicated a few white powdery spots (1–33% leaves infected), 2 indicated a few more white powdery spots (33–66% leaves infected), 3 indicated significantly more white powdery spots (66–80% leaves infected), 4 indicated almost the whole leaf covered by disease spots with slight necrosis (> 80% leaves infected), and 5 indicated the whole plant leaves covered entirely with disease spots and serious yellowing (**Supplementary Figure 1**). The DSI for each of the 331 SGAs was calculated using the formula: DSI = (Σ (rating of each plant)/5×total numbers of plants rated) × 100. The DSI scale ranges from 0 to 100, where 0 represents no disease symptoms and 100 represents complete fungal coverage. Based on DSI values, the PMD resistance of all SGAs was classified as highly resistant (HR, DSI < 5.00), resistant (R, DSI 5.01–15.00), moderately resistant (MR, DSI 15.1–30.00), moderately susceptible (MS, DSI 30.01–50.00), susceptible (S, DSI 50.01–70.00), or highly susceptible (HS, DSI > 70.01).

### 5.3 DNA extraction, genotyping, and quality control

Genomic DNA was extracted from fresh young soybean leaves using the hexadecyl trimethyl ammonium bromide method as previously published (Kisha et al., 1997). Genotyping of the 331 SGAs was performed with the Illumina SoySNP50k iSelect BeadChip (Illumina, San Diego, Calif. USA), which included 52,041 SNPs (Song et al., 2013). Using the GenomeStudio Genotyping Module v1.8.4 (Illumina, Inc., San Diego, CA), the SNP alleles were called. A total of 42,509 SNP loci were successfully obtained, where 429 SNP loci were not mapped to the 20 soybean genomes. Therefore, 42080 SNP loci were used as genotypic data in this study.The SNP data were represented using the International Union of Pure and Applied Chemistry standard codes for nucleotides. Each SNP marker’s quality was individually assessed following previous reports (Yan et al., 2010). SNPs without physical position information and displaying low quality (missing data < 20% and/or minor allele frequency (MAF) < 0.05) across all samples were excluded from the dataset. The remaining 30,602 high-quality SNP markers were retained for further analysis.

### 5.4 Population structure and LD

Population stratification was inferred using PCA, neighbor-joining (NJ) phylogenetic trees, and population structure analysis. Tassel V5.2.60 was employed for PCA and kinship matrix calculations based on 30,602 SNPs from the 331 SGAs, where the kinship Matrix_Type was Centered_IBS. The NJ tree was constructed using the Maximum Composite likelihood model in MEGA-X, in which the Bootstrap value was 1000 replicates, the Gaps/Missing Data Treatment selected partial deletion, and the Site Coverage Cutoff was 80%. Linkage SNP filtering was performed using PLINK V1.09, with a window size of 50 kb, SNP step size of 10, SNP correlation threshold of 0.2, and retention of unlinked SNPs, resulting in 1643 SNPs for population structure inference using STRUCTURE 2.3.4 (Pritchard et al., 2000). The number of subgroups (K) was set from 1 to 10 with 5 replications. The length of the burn-in period was set to 10,000, and the number of Monte Carlo Markov Chain replications was set to 100,000, with other options using the default values of the software. The most likely K value was determined using Structure Harvester (Earl and Vonholdt, 2012) based on Delta K (Evanno et al., 2005). Pairwise LD (MAF< 0.05) estimation was conducted on 30,602 SNPs using squared allele frequency correlations (r^2^) with PLINK1.09. Mean LD decay plots were generated using an R script (Remington et al., 2001), plotting r^2^ values for SNPs within 1000 kb pairwise distances against the physical distance on each chromosome. The LD decay rate was determined as the chromosomal physical distance at which the mean r^2^ decreased to half its maximum value (Huang et al., 2010). The LD analysis and identification of haplotype blocks for significant SNPs were conducted utilizing LDBlockShow Software (Dong et al., 2021).

### 5.5 GWAS

Missing SNP genotypes in the filtered dataset were imputed using Beagle software (Browning et al., 2021). A total of 30,602 SNP markers from 331 SGAs were employed to detect association signals between the SNPs and DSI. The GWAS analysis utilized GAPIT with MLM (Yu et al., 2006) and CMLM (Lipka et al., 2012), as well as FaST-LMM (Lippert et al., 2011) and EMMAX (Kang et al., 2010). The analysis included a reduced population structure matrix (Q) and a kinship matrix as covariates for population structure and familial relatedness, respectively. Significant association signals were identified using the Bonferroni threshold, with a threshold set at P ≤ 1/30,602, or -Log10(P) ≥ 4.49 (Li et al., 2019).

### 5.6 Candidate gene prediction and qRT-PCR assay

We concentrated on significant SNPs associated with large-effect quantitative trait nucleotides and performed a targeted search within their genomic regions to identify candidate genes generating the causal signals. Candidate physical regions were defined based on either the mean LD decay distance or the LD block. Gene identification was achieved by obtaining functional annotations of gene models (Glyma.Wm82.a2.v1) or known genes within the target genomic regions from the Soybase Database (http://www.soybase.org/). Utilizing soybean genome annotations, we predicted putative causal genes associated with the identified regions. Furthermore, functional annotations of genes in the target genomic regions were retrieved from Phytozome (http://www.phytozome.net). For the gene expression analysis, the *M. diffusa* spore suspension (1×10^5^ cfu/ml) was sprayed onto seedlings of ZDD06944 (HS, DSI=100.0) and ZDD00359 (HR, DSI=0.0) after they had been grown for 10 d. The seedlings were then stored in a growth chamber at 75% relative humidity, 23°C, and a photoperiod of 16 h of light and 8 h of darkness. Primary leaves were sampled at 0, 6, 12, 24, 48, and 72 h after inoculation, and total RNA was extracted using an Easy Pure Plant RNA kit (QUANSHIJIN, China). Reverse transcription was performed on 1.5 μg of DNase-treated RNA utilizing a PrimeScript™ RT Reagent kit with gDNA Eraser from Takara (Japan). The qRT-PCR primers were designed using the Oligo7 software (**Supplementary Table S7**), and the housekeeping gene actin was selected as the control gene. The qRT-PCR analyses were applied to identify the expression level of each candidate’s PMD resistance gene. Real-time RT-PCR amplifications were performed on the CFX48 ECO™ Real-Time PCR System (Illumina, USA) utilizing the RT-PCR kit according to the manufacturer’s instructions (Takara, Japan). The qRT-PCR reaction was prepared by combining 0.2 µM primer premix, 5 µL TB Green Premix Ex Taq II (TaKaRa, Japan), 2 µL of cDNA synthesis solution, and using ultra-pure water to adjust the final volume to 10 µL. The qRT-PCRs were performed as follows: 50°C for 2 min, 95°C for 3 min, followed by 40 cycles, 95°C for 10 s, 50 or 61°C (associated with the gene), and 72°C for 30 s. In order to ensure reliable statistical analysis, three independent biological replicates were conducted, and the comparative 2^−ΔΔCt^ method was adopted to evaluate the relative expression levels of the candidate genes (Livak and Schmittgen, 2001).

## Data availability statement

The SNP data used in this study have been deposited and published in the China National GeneBank DataBase (CNGBdb), accession number: CNP0004650; see the following link for details: https://db.cngb.org/search/project/CNP0004650/.

## Author contributions

YS: Data curation, Formal Analysis, Investigation, Software, Writing – original draft. HZ: Funding acquisition, Writing – review & editing. XL: Project administration, Writing – review & editing. CY: Writing – review & editing. GQ: Investigation, Writing – review & editing. YL: Writing – review & editing. LD: Investigation, Writing – review & editing. YNW: Investigation, Writing – review & editing. DW: Conceptualization, Resources, Writing – review & editing. YMW: Conceptualization, Supervision, Validation, Writing – review & editing. YD: Conceptualization, Funding acquisition, Project administration, Supervision, Validation, Visualization, Writing – review & editing.

## Supplementary material

Supplementary Figure 1 Soybean PMD scoring scheme. **(A–F)** displayed phenotypes representing different disease severity scores, ranging from 0 to 5.
