# Supplementary figures and images for: Genome-wide association study of powdery mildew resistance in cultivated soybean from Northeast China
Source: Front Plant Sci. 2023 Nov 2;14:1268706. doi: 10.3389/fpls.2023.1268706 (PMC10651740; doi:10.3389/fpls.2023.1268706)

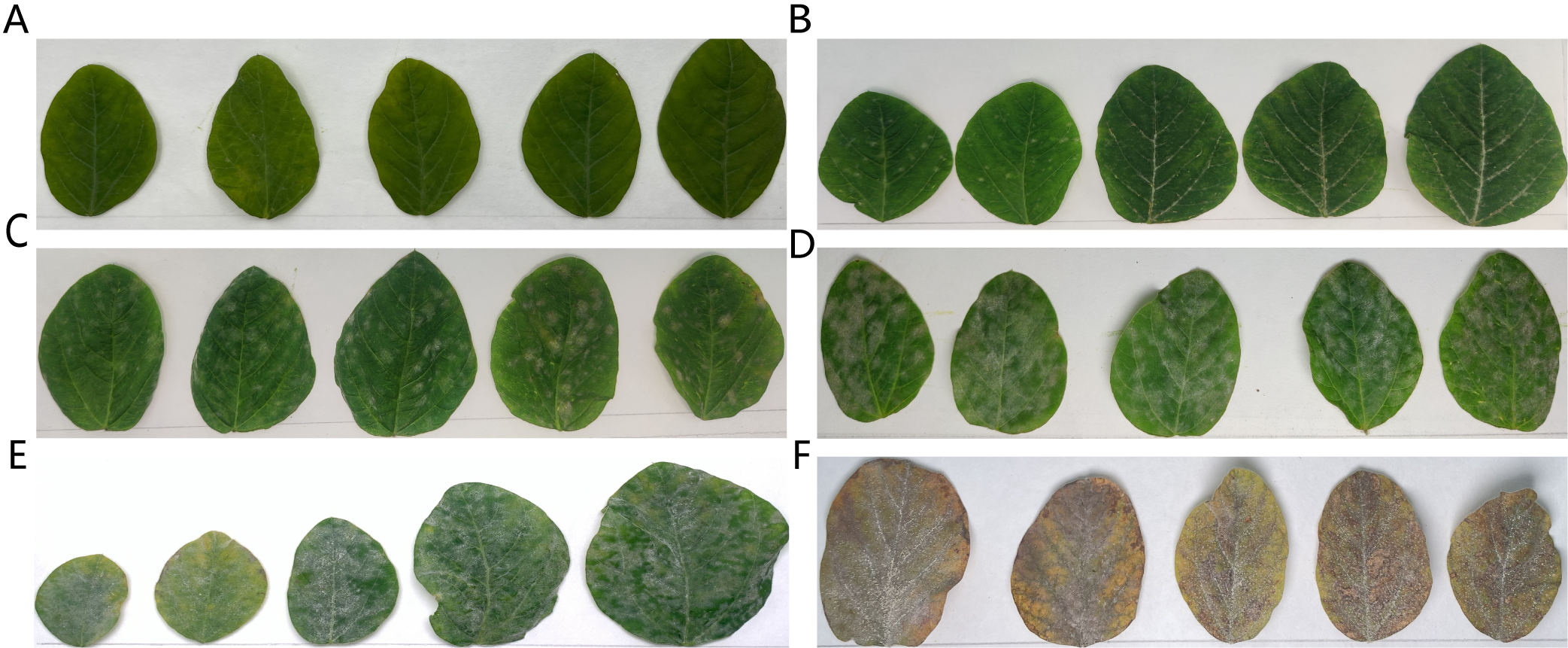

Supplement: Supplementary Figure 1 — Soybean PMD scoring scheme. (A–F) displayed phenotypes representing different disease severity scores, ranging from 0 to 5. [file Image_1.tif]
